# A Leech Capable of Surviving Exposure to Extremely Low Temperatures

**DOI:** 10.1371/journal.pone.0086807

**Published:** 2014-01-22

**Authors:** Dai Suzuki, Tomoko Miyamoto, Takahiro Kikawada, Manabu Watanabe, Toru Suzuki

**Affiliations:** 1 Department of Food Science and Technology, Tokyo University of Marine Science and Technology, Tokyo, Japan; 2 Department of Zoology, Graduate School of Science, Kyoto University, Kyoto, Japan; 3 Anhydrobiosis Research Group, Insect Mimetics Research Unit, National Institute of Agrobiological Sciences, Tsukuba, Japan; Iwate University, Japan

## Abstract

It is widely considered that most organisms cannot survive prolonged exposure to temperatures below 0°C, primarily because of the damage caused by the water in cells as it freezes. However, some organisms are capable of surviving extreme variations in environmental conditions. In the case of temperature, the ability to survive subzero temperatures is referred to as cryobiosis. We show that the ozobranchid leech, *Ozobranchus jantseanus*, a parasite of freshwater turtles, has a surprisingly high tolerance to freezing and thawing. This finding is particularly interesting because the leach can survive these temperatures without any acclimation period or pretreatment. Specifically, the leech survived exposure to super-low temperatures by storage in liquid nitrogen (−196°C) for 24 hours, as well as long-term storage at temperatures as low as −90°C for up to 32 months. The leech was also capable of enduring repeated freeze-thaw cycles in the temperature range 20°C to −100°C and then back to 20°C. The results demonstrated that the novel cryotolerance mechanisms employed by *O. jantseanus* enable the leech to withstand a wider range of temperatures than those reported previously for cryobiotic organisms. We anticipate that the mechanism for the observed tolerance to freezing and thawing in *O. jantseanus* will prove useful for future studies of cryopreservation.

## Introduction

In most ectothermic organisms, prolonged exposure to temperatures below 0°C can cause the water in their tissues to freeze, resulting in permanent physiological damage and sometimes cell death. However, organisms that live in extreme environments, such as polar regions or mountain highlands, frequently endure subzero temperatures [1]–[3]. Organisms in temperate zones typically survive such temperatures by hibernating [4], [5]. Cryobiosis, which refers to the adaptation or tolerance to freezing temperatures, is a form of cryptobiosis [6]. Among ectotherms, freeze tolerance should only be used to describe an ecologically relevant hibernation strategy that includes the ability to survive long-term freezing while maintaining constant and maximal intracellular ice contents at the subzero temperatures that are naturally encountered in the hibernaculum [7].

**Figure 1:**
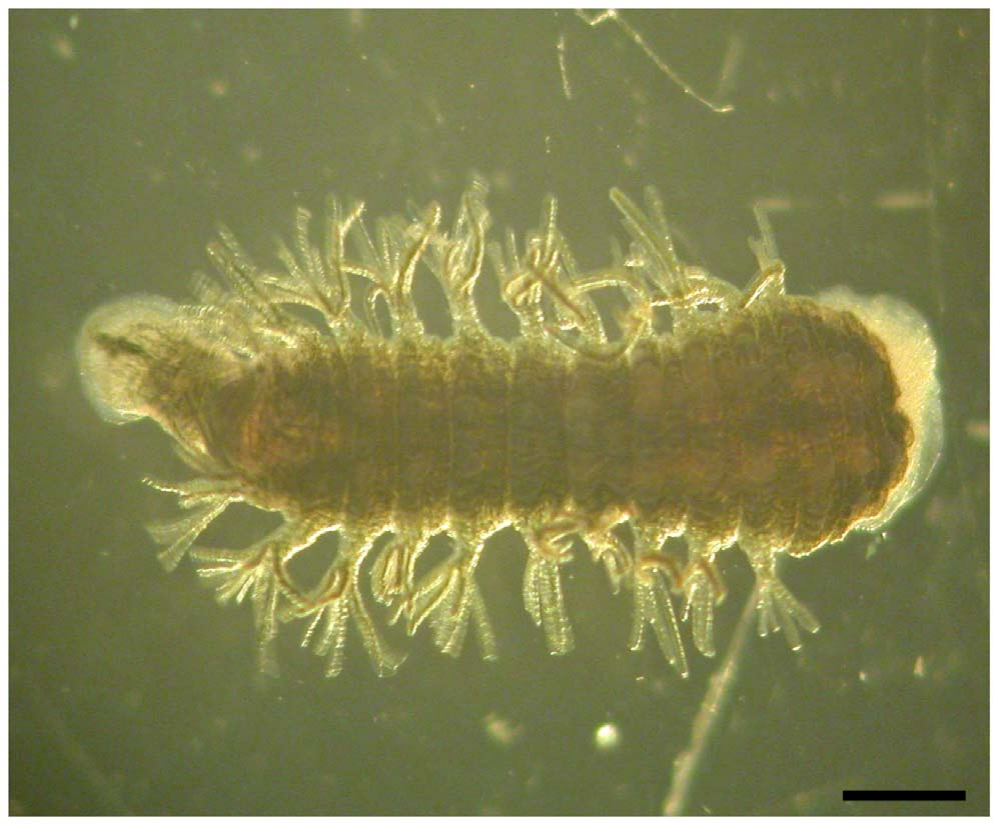
Stereoscopic micrograph of *Ozobranchus jantseanus* (dorsal view). Scale bar = 1 mm.

The leech genus *Ozobranchus* (Annelida: Hirudinida: Ozobranchidae) contains seven species that parasitize turtles [8]. *Ozobranchus jantseanus* is an external parasite on the freshwater turtles *Mauremys japonica* and *M. reevesii*, and relies on its turtle hosts for all of its life stages, i.e. from the egg to the adult stage [9] (Fig. 1). The leech is distributed throughout East Asia, Japan and China [9].

Several turtle species experience subzero temperatures (−2 to −4°C) for 1 to 11 days in their hibernacula [10], and *M. japonica* and *M. reevesii*, the hosts of *O. jantseanus*, typically hibernate in environments where they are likely to periodically encounter temperatures as low as 0°C. However, it would be unusual for both the leeches and their turtle hosts to exist under such cold conditions for extended periods of time.

In the present study, we examined cryoresistance in *O. jantseanus.* The results showed that the leech was capable of surviving exposure to extremely low temperatures (−196°C) as well as prolonged storage at −90°C. All of the individuals had been active before initiating the experiments, indicating that this cryoresistance ability is always present in *O. jantseanus.*

## Results and Discussion

**Figure 2:**
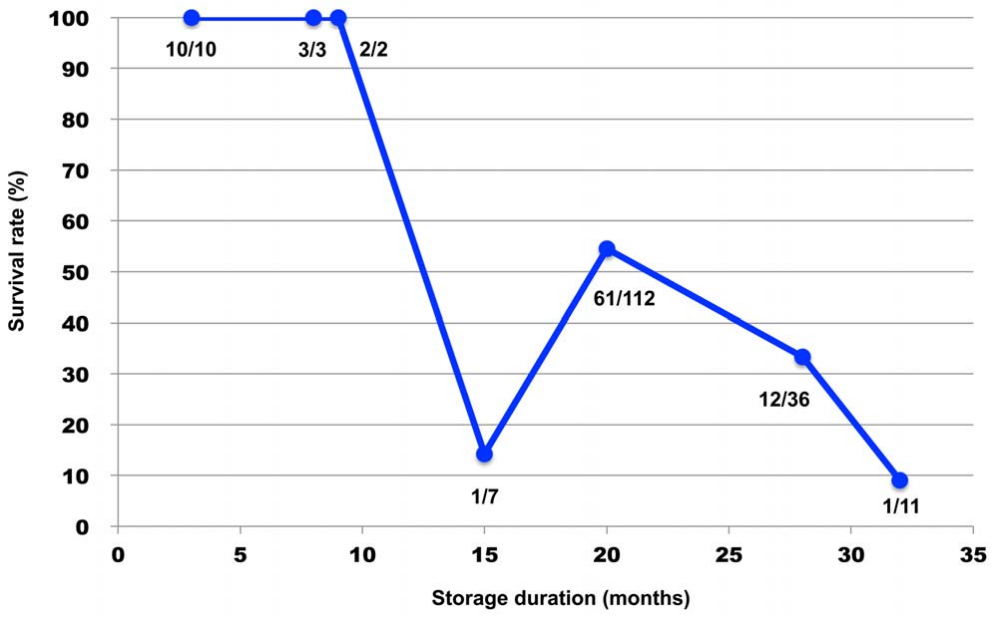
Survival rates of *Ozobranchus jantseanus* after long-term storage at −90°C. Values below data points refer to the number of individuals subjected to storage. Leeches were considered to be alive if they moved within five hours of thawing.

**Figure 3:**
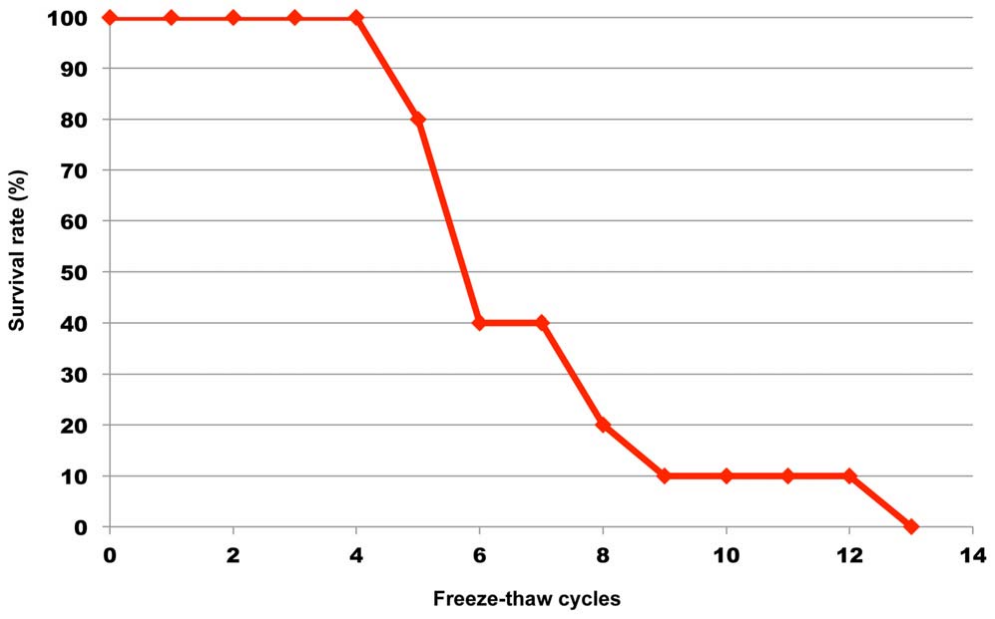
Survival rates of *Ozobranchus jantseanus* after repeated freeze-thaw cycles. Freeze-thaw cycles determined by differential scanning colorimetry consisted of cooling from 20°C to −100°C and then thawing to 20°C at 10°C/min.

**Figure 4:**
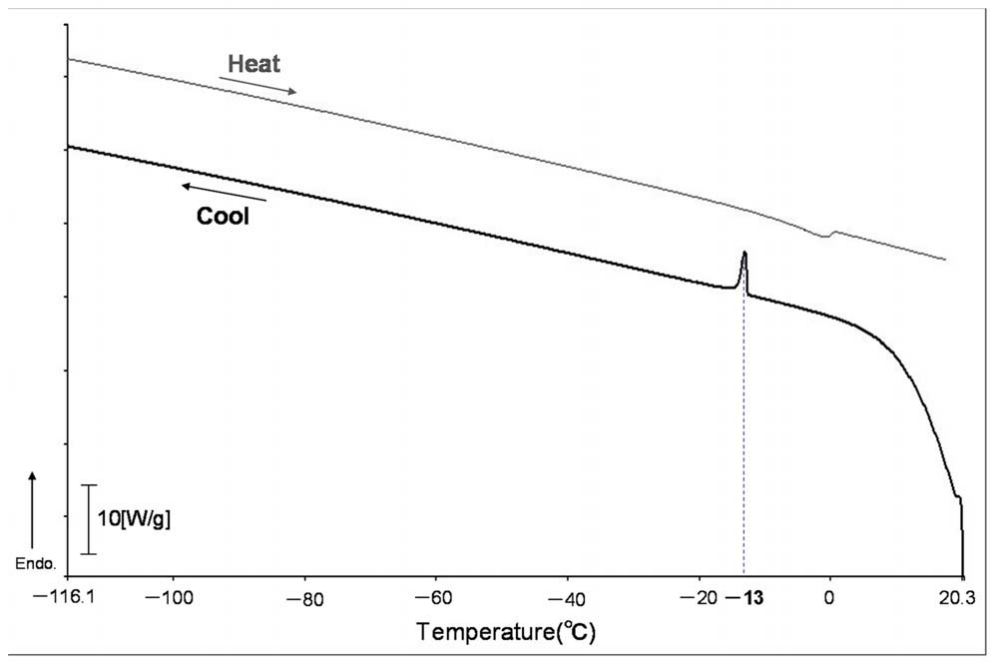
Thermal analysis of *Ozobranchus jantseanus* by differential scanning colorimetry for temperatures 20°C to −120°C and then back to 20°C at ±10°C/min.

Of the seven leech species that were subjected to cryoexposure tests at −90°C or −196°C for 24 hours, only *O. jantseanus* survived both temperatures (Table 1). Since all of the other leech species were not capable of surviving exposure even to −90°C, the resistance to freezing exhibited by *O. jantseanus* appeared to be unique. Subjecting the leeches to repeated freeze-thaw cycles spanning several minutes revealed that they were able to survive extreme fluctuations in temperature without employing any means of temperature acclimation. The thawed adult leeches that cooled down to −90°C or −196°C survived for maximum of 45 or 39 days in distilled water without feeding, respectively. The reason why the leeches died is considered mainly by starving rather than by freezing injury. However, the little difference of survival time between −90°C and −196°C seemed to be caused by the difference in the degree of freezing injury. In addition, because the four *O. jantseanus* hatchlings had not been provided with a blood meal before exposure to −90°C, the temperature tolerance observed in *O. jantseanus* is considered to be innate and not attributable to ingesta. It was also found that *O. jantseanus* become active again after long-term exposure to temperatures as low as −90°C for up to 32 months (Fig. 2). All of the individuals stored at −90°C for three, eight, and nine months survived, but survival began to decrease after 15 months of storage. Despite the gradual increase in mortality observed as the duration of low-temperature storage increased, these results showed that *O. jantseanus* was capable of surviving long-term cold storage (Fig. 2). One of the reasons why the survival rates of the leech in the storage decreased may be caused by the biological activity, such as an enzymatic reaction, remaining at cold temperature [11]. We also showed that *O. jantseanus* was capable of surviving repeated freeze-thaw cycles (Fig. 3). Specifically, leeches survived an average of four freeze-thaw cycles, but a maximum of 12 freeze-thaw cycles was recorded (Fig. 3). In addition, thermal analysis using differential scanning calorimetry (DSC) showed that an exothermic peak formed at approximately −13°C in the cooling cycle (Fig.4), implying that leeches appeared to freeze through experience of supercooling. However, we were unable to determine whether freezing was intracellular or extracellular, and glass transition could not be observed from −120°C to 20°C. Although the stress associated with freezing and thawing is expected to be deleterious to the leeches, exposure to a several freeze-thaw cycles does not appear to be fatal. Nonetheless, the activity and behavior of *O. jantseanus* after thawing did differ markedly from their behavior before freezing, i.e. they shrank and appeared to be unable to elongate their body. Therefore, it suggested that the leech might be gradually damaged by the freeze-thaw stresses. Furthermore, it was also unclear how characteristics such as feeding and reproductive capacity differed between leeches that had undergone repeated freeze-thawing and those that had not. However, since the thawed leeches actively moved their bodies and gills, they were considered to have survived the stresses associated with freezing and thawing.

**Table 1:** Survival rates of seven leech species after storage in a deep freezer (−90°C; all species) and liquid nitrogen (−196°C; *Ozobranchus jantseanus* only) for 24 hours.

Species	Temperature (°C)	Total no. individuals	Survival rate (%)
*Ozobranchus jantseanus* (adult)	−90	5	100
*O. jantseanus* (hatchling)	−90	4	100
*O. margoi*	−90	10	0
*Glossiphonia complanata*	−90	1	0
*Alboglossiphonia lata*	−90	2	0
*Helobdella stagnalis*	−90	5	0
*Erpobdella octoculata*	−90	5	0
*E. japonica*	−90	4	0
*O. jantseanus* (adult)	−196	5	100

Several organisms have been reported to survive freezing through a process referred to as cryptobiosis, and some nematode species are known to be resistant to freezing [12]. Of these, the Antarctic nematode, *Panagrolaimus davidi*, has been reported to be particularly cold resistant, with 23.8% of nematodes surviving 28 days exposure to −80°C [13]. Nonetheless, survival in *P. davidi* was markedly lower than that observed for *O. jantseanus* in this study, with 100% of *O. jantseanus* surviving exposure to −90°C for 9 months (see Fig. 2).

Of the organisms that have been experimentally subjected to very low temperatures, only the Tardigrade, *Ramazzottius varieornatus*, and the diapause larva of the drosophilid fly, *Chymomyza costata*, have been reported to survive immersion in liquid nitrogen, i.e. −196°C [14], [15]. However, the durations of the cryoexposure tests were only 15 min and 1 hour for *R. varieornatus* and *C. costata*, respectively, which was shorter than the 24-hour storage period employed in this study (see Table 1). In addition, the survival rate of *R. varieornatus* stored in liquid nitrogen was low (22.3%). The survival of diapause-destined *C. costata* larvae, or warm-acclimated larvae, was lower than cold-acclimated specimens, and specimens that were not in diapause did not survive storage in liquid nitrogen [15].

Anhydrobiosis, which is a type of cryptobiosis, refers to an extremely dehydrated state in which the organism does not exhibit any metabolic activity, but which retains the ability to revive itself after rehydration [16]. Interestingly, these anhydrobiotes are also capable of cryobiosis [17]. In fact, anhydrobiotic organisms, such as the tardigrades and the sleeping chironomid, *Polypedilum vanderplanki*, can survive ultralow temperatures (e.g. –196°C) [14], [16], [17]. It has been shown that this condition is associated with vitrification, which is facilitated by the accumulation of large amounts of disaccharides, such as a trehalose [18]. In addition, the dehydration of organs also facilitates survival by reducing the amount of extracellular ice that can form in the interstices between cells, thereby limiting physical damage by ice [7]. However, entering this anhydrobiotic stage requires a period of acclimation, for example 48 hours in the sleeping chironomid [16], during which the fly dehydrates itself and synthesizes the cryoprotectants (e.g. trehaloses) that it needs to survive the physiological stresses associated with a dry environment.

Since the *O. jantseanus* in this study were exposed to extremely low temperatures within a very short time period, the time required to initiate the metabolic pathways required for cryoprotection is considered to have been insufficient. Indeed, trehalose or glycerol, which are typical cryoprotectants, were not detected before or after freezing (data not shown), implying that the low-temperature tolerance observed in the leeches of this study is not attributable to these saccharides; instead, it appears that that the leeches may be capable of tolerating physiological water freezing in their tissues. Anhydrobiotic organisms can withstand low temperatures indefinitely by becoming dehydrated, but cryobiotic organisms do not employ desiccation [17]. Indeed, the finding that *O. jantseanus* cannot survive dry conditions, for example a keeping of the leech at a petri dish without water in 24 hours at room temperature, implies that this leech species is a cryobiotic organism. Further, the observation that three of the thawed eggs hatched after storage at −90°C for 24 hours and that leeches did not need to acclimatize to low temperatures implied that the leeches have an innate capacity for withstanding such cold stresses. It has also been reported that, under the right conditions, cells and organisms can be cryopreserved at very low temperatures, even if they have no natural capacity for surviving low temperatures (e.g., mouse embryos [19], red blood cells [20]). However, unlike *O. jantseanus*, the ability of these specimens to survive exposure to low temperatures is not considered to be due to some innate ability.

It is unlikely that *O. jantseanus* would encounter similar freeze-thaw cycles in its natural environment of [9], [21], [22], so it is suggested that the cold tolerance observed in this species has not arisen in response to some ecological need or that it is an environmental adaptation. Rather, it is likely that this cryotolerant ability has arisen in response to some as yet unclarified adaptation. We propose that, compared to other cryobiotic organisms, *O. jantseanus* exhibits the most robust cryotolerance ability reported to date. It is hoped that these findings will contribute to the development of new cryopreservation methods that do not require additives, and also to the resuscitation of organisms that have been frozen underground in permafrost areas, on Antarctica, and possibly on other planets.

## Materials and Methods

A total of 205 *O. jantseanus* individuals were collected from their hosts, the freshwater turtles *M. japonica* or *M. reevesii*, which were captured at two sites in the Kyoto and Chiba prefectures of Japan. A further six leech species were collected for comparative purposes: one marine species that is a parasite on sea turtles, *O. margoi* (n = 10), and five nonparasitic, freshwater species, *Glossiphonia complanata* (n = 1), *Alboglossiphonia lata* (n = 2), *Helobdella stagnalis* (n = 5), *Erpobdella octoculata* (n = 5), and *E. japonica* (n = 4). The *O. margoi* specimens were collected from a loggerhead turtle, *Caretta caretta*, along the Muroto coast of Japan, and the freshwater species (including *O. jantseanus*) were collected in the Kamogawa River in Kyoto Prefecture, Japan. The Guidelines for Animal Experiments at Tokyo University of Marine Science and Technology were employed in this study, and the study was approved by Professor Nobuaki Okamoto, President of Tokyo University of Marine Science and Technology. The Guidelines for Animal Experiments at Tokyo University of Marine Science and Technology are based on the Guidelines for Animal Experiments that were promulgated by the Science Council of Japan and the Ministry of Education, Culture, Sports, Science and Technology, Japan on June 1, 2006. The ozobranchid leeches were removed from the turtles using forceps, and the other leech species were collected from stones on the riverbed. The leech species and freshwater turtles are not subject to any animal protection laws in Japan and no permits were required to handle them and/or capture them by hand or by using cage traps. The use of traps is not illegal at the sampling sites in this study, and the turtles and the leeches were not injured during sampling. Freshwater turtles were released after the leeches were collected. The sea turtle, which is a protected species, was caught accidentally as fishing by-catch and was not injured by the collection of leeches. The turtle was released back into the sea once the leeches were removed.

Cold tolerance in adults (total length 1–15 mm), hatchlings (total length: <1 mm), and eggs (containing approximately 20 eggs and the size of an egg was about 0.75 mm×0.75 mm) of *O. jantseanus*, was examined by placing the leeches in a deep freezer (−90°C) or in liquid nitrogen (−196°C; only adult *O. jantseanus*) for 24 hours. For adult leech of other species (total length about 5–50 mm), the same cooling tests by a deep freezer were conducted. Prior to freezing, all individuals were maintained in either freshwater or seawater at room temperature, and water on their surface was wiped just before the freezing examination. For the deep freezer test, the samples were put into a thin plastic film bag individually or one cocoon, and the bag was put into immediately the deep freezer where was kept at −90°C. On the other hand, for the liquid nitrogen immersion test, the samples were directly immersed individually. The exact cooling rates of specimens could not be measured since leech was so small and the weight was less than 20 mg. However, the actual cooling times to attain the purpose temperature −90°C or −196°C were at least within 10 seconds. Essentially, the purpose of this examination was to know the effect of end-point temperature. After the cryoexposure tests, the frozen leeches were pic up from the bag and thawed by immersion in either distilled water or seawater at room temperature. In this thawing, also the speeds were not measured, but the thawing to room temperature was finished within a few second. After thawing, specimens that restarted moving again under their own power (such as action of the gills and shrinking of the body) within five hours were judged as being alive. In the case of eggs, we thawed the frozen cocoons by storing in a petri dish containing distilled water, and kept them in the dish for a week. As the leeches hatched out from the cocoons that experienced eh cryoexposure test, we considered the eggs to have the cryotolerance.

To clarify long-term freeze tolerance in *O. jantseanus*, the survival rates of leeches stored at −90°C for 3 to 32 months were compared. The number of individuals and duration of freezing were 10, 3, 2, 7, 112, 36, and 11 leeches, and 3, 8, 9, 15, 20, 28, and 32 months, respectively. The freezing and thawing methods, and the judgment of survival were same to above test.

Using differential scanning calorimetry (DSC; Shimadzu DSC-50, Japan), we also counted how many freeze-thaw cycles adult *O. jantseanus* specimens were capable of surviving. Ten adult individuals of the leech were put into aluminum DSC cell without seal, and were set on DSC. A freeze-thaw cycle test was conducted as following protocol; the sample were cooled down at −10°C/min from 20°C to −100°C, immediately without holding at −100°C, and warmed at +10°C/min to 20°C. The survival at each cycle was judged from whether they started moving or not within 20 minutes after reached to 20°C. After assessing survival in this way, the freeze-thaw process was repeated until all of the specimens were classified as dead. We also examined the physicochemical characteristics such as freezing points, glass transition by DSC. The one individual was put into aluminum DSC cell and hermetically sealed. Samples were cooled down from 20°C to −120°C and warmed to 20°C at ±10°C/min. Prior to this experiment, any water on the body surface of the leeches was wiped and removed using an air dryer to exclude the effect of water out of body which may affect DSC results by their freezing.

## References

[1] Kohshima S (1984) A novel cold-tolerant insect found in a Himalayan glacier. Nature 310: 225.

[2] Sømme L, Meier T (1995) Cold tolerance in Tardigrada from Dronning Maud Land, Antarctica. Polar Biol 15: 221–224.

[3] Rothschild LJ, Mancinelli RL (2001) Life in extreme environments. Nature 409: 1092–1101. 11234023 10.1038/35059215

[4] Diamond JM (1989) Resurrection of frozen animals. Nature 339: 509–510.

[5] Storey KB, Storey JM (1996) Natural freezing survival in animals. Annu Rev Ecol Syst 27: 365–386.

[6] Keilin D (1959) The problem of anabiosis or latent life: history and current concept. Proc R Soc Lond B 150: 149–191. 13633975 10.1098/rspb.1959.0013

[7] Storey KB, Storey JM (1992) Natural freeze tolerance in ectothermic vertebrates. Annu Rev Physiol 54: 619–637. 1562185 10.1146/annurev.ph.54.030192.003155

[8] Sawyer RT (1986) Leech Biology and Behaviour. Oxford: Clarendon Press. 1065 p.

[9] Yamauchi T, Suzuki D (2008) Geographic distribution of *Ozobranchus jantseanus* (Annelida: Hirudinida: Ozobranchidae) in Japan. Med Entomol Zool 59: 345–349.

[10] Storey KB (2006) Reptile freeze tolerance: Metabolism and gene expression. Cryobiol 52: 1–16. 10.1016/j.cryobiol.2005.09.005 16321368

[11] Agustini TW, Suzuki T, Hagiwara T, Ishizaki S, Tanaka M, et al (2001) Change of *K* value and water state of yellowfin tuna *Thunnus albacares* meat stored in a wide temperature range (20°C to −80°C). Fish Sci 67: 306–313.

[12] Smith T, Wharton DA, Marshall CJ (2008) Cold tolerance of an Antarctic nematode that survives intracellular freezing: comparisons with other nematode species. J Comp Physiol B 178: 93–100. 17712562 10.1007/s00360-007-0202-3

[13] Wharton DA, Brown IM (1991) Cold-tolerance mechanisms of the Antarctic nematode *Panagrolaimus davidi* . J Exp Biol 155: 629–641. 10.1242/jeb.00083 12477892

[14] Horikawa DD, Kunieda T, Abe W, Watanabe M, Nakahara Y, et al (2008) Establishment of a rearing system of the extremotolerant tardigrade *Ramazzottius variornatus*: a new model animal for astrobiology. Astrobiol. 8: 549–556. 10.1089/ast.2007.0139 18554084

[15] Koštál V, Zahradníčková H, Šimek P (2011) Hyperprolinemic larvae of the drosophilid fly, *Chymomyza costata*, survive cryopreservation in liquid nitrogen. Proc Natl Acad Sci U S A 108: 13041–13046. 21788482 10.1073/pnas.1107060108 PMC3156168

[16] Sakurai M, Furuki T, Akao K, Tanaka D, Nakahara Y, et al (2008) Vitrification is essential for anhydrobiosis in an African chironomid, *Polypedilum vanderplanki* . Proc Natl Acad Sci U S A 105: 5093–5098. 18362351 10.1073/pnas.0706197105 PMC2278217

[17] Wright JC (2001) Cryptobiosis 300 years on from van Leeuwenhoek: What have we learned about Tardigrades? Zool Anz 240: 563–582.

[18] Crowe JH, Carpenter JF, Crowe LM (1998) The role of vitrification in anhydrobiosis. Annu Rev Physiol 60: 73–103. 9558455 10.1146/annurev.physiol.60.1.73

[19] Rall WF, Fahy GM (1985) Ice-free cryopreservation of mouse embryos at −196°C by vitrification. Nature 313: 573–575. 3969158 10.1038/313573a0

[20] Rowe AW, Eyster E, Kellner A (1968) Liquid nitrogen preservation of red blood cells for transfusion: A low glycerol-Rapid freeze procedure. Cryobiol. 5: 119–128. 10.1016/s0011-2240(68)80154-3 5717951

[21] Yasukawa Y, Yabe T, Ota H (2008) *Mauremys japonica* (Temminck and Schlegel, 1835) – Japanese pond turtle. *Chelonian Res Monogr* 5: 003.1–003.6.

[22] Lovich JE, Yasukawa Y, Ota H (2011) *Mauremys reevesii* (Gray 1831) – Reeves’ turtle, Chinese three-keeled pond turtle. Chelonian Res Monogr 5: 050.1–050.10.

